# Co-authorship Network Analysis: A Powerful Tool for Strategic Planning of Research, Development and Capacity Building Programs on Neglected Diseases

**DOI:** 10.1371/journal.pntd.0000501

**Published:** 2009-08-18

**Authors:** Carlos Medicis Morel, Suzanne Jacob Serruya, Gerson Oliveira Penna, Reinaldo Guimarães

**Affiliations:** 1 National Institute for Science and Technology on Innovation on Neglected Diseases (INCT/IDN), Center for Technological Development in Health (CDTS), Oswaldo Cruz Foundation (Fiocruz), Rio de Janeiro, Brazil; 2 Department of Science and Technology (DECIT), Secretary of Science, Technology and Strategic Goods (SCTIE), Ministry of Health, Brasilia, Brazil; 3 Secretary of Health Surveillance (SVS), Ministry of Health, Brasilia, Brazil; 4 Secretary of Science, Technology and Strategic Goods (SCTIE), Ministry of Health, Brasilia, Brazil; Swiss Tropical Institute, Switzerland

## Abstract

**Background:**

New approaches and tools were needed to support the strategic planning, implementation and management of a Program launched by the Brazilian Government to fund research, development and capacity building on neglected tropical diseases with strong focus on the North, Northeast and Center-West regions of the country where these diseases are prevalent.

**Methodology/Principal Findings:**

Based on demographic, epidemiological and burden of disease data, seven diseases were selected by the Ministry of Health as targets of the initiative. Publications on these diseases by Brazilian researchers were retrieved from international databases, analyzed and processed with text-mining tools in order to standardize author- and institution's names and addresses. Co-authorship networks based on these publications were assembled, visualized and analyzed with social network analysis software packages. Network visualization and analysis generated new information, allowing better design and strategic planning of the Program, enabling decision makers to characterize network components by area of work, identify institutions as well as authors playing major roles as central hubs or located at critical network cut-points and readily detect authors or institutions participating in large international scientific collaborating networks.

**Conclusions/Significance:**

Traditional criteria used to monitor and evaluate research proposals or R&D Programs, such as researchers' productivity and impact factor of scientific publications, are of limited value when addressing research areas of low productivity or involving institutions from endemic regions where human resources are limited. Network analysis was found to generate new and valuable information relevant to the strategic planning, implementation and monitoring of the Program. It afforded a more proactive role of the funding agencies in relation to public health and equity goals, to scientific capacity building objectives and a more consistent engagement of institutions and authors from endemic regions based on innovative criteria and parameters anchored on objective scientific data.

## Author Summary

The selection and prioritization of research proposals is always a challenge, particularly when addressing neglected tropical diseases, as the scientific communities are relatively small, funding is usually limited and the disparity between the science and technology capacity of different countries and regions is enormous. When the Ministry of Health and the Ministry of Science and Technology of Brazil decided to launch an R&D program on neglected diseases for which at least 30% of the Program's resources were supposed to be invested in institutions and authors from the poorest regions of Brazil, it became clear to us that new strategies and approaches would be required. Social network analysis of co-authorship networks is one of the new approaches we are exploring to develop new tools to help policy-/decision-makers and academia jointly plan, implement, monitor and evaluate investments in this area. Publications retrieved from international databases provide the starting material. After standardization of names and addresses of authors and institutions with text mining tools, networks are assembled and visualized using social network analysis software. This study enabled the development of innovative criteria and parameters, allowing better strategic planning, smooth implementation and strong support and endorsement of the Program by key stakeholders.

## Introduction

The World Health Organization (WHO) classifies diseases as Type I, Type II and Type III, which largely corresponds to Global, Neglected and Most Neglected diseases in the vocabulary of the international organization *Medécins Sans Frontières* (MSF) [1],[2]. Type I/Global diseases know no geographic boundaries while Type II–III/Neglected-Most Neglected are predominantly or exclusively prevalent among populations of developing countries. Types II and III diseases (from now on “neglected diseases”), being prevalent in poor regions, are not prioritized by pharmaceutical and biotechnological industries responsible for the manufacture of goods such as vaccines, drugs and diagnostic kits. This generates what is known as ‘market failures’ - the inefficient allocation of products and services through usual free market mechanisms.

Several procedures have been suggested to cope with the three types of “health failures”: (i) *Science failures* (insufficient knowledge prevents the development of health products such as malaria and HIV vaccines): Stimulate basic, fundamental research and technological development, (ii) *Market failures* (high prices prevent access of drugs by needy populations): Price reduction policies (resulting e.g. from negotiations between governments and industry) or creating subsidizing mechanisms leading to lower prices and (iii) *Health service failures* (inexpensive drugs do not reach the patients): Fighting corruption, reducing inequalities and coping with cultural, religious or infrastructure barriers, etc. that prevent access to cheap or free drugs by poor countries [3],[4].

Several initiatives have recently been proposed to stimulate research, technological development and production of vaccines, drugs and diagnostics for neglected diseases by both Big Pharma and Small Biotech of developed countries such as “Push” mechanisms, like Public Private Partnerships (PPPs) or Partnerships for the Development of Products (PDPs), funded in general by philanthropies or governments [5],[6] and “Pull” mechanisms, like Advance Market Commitments (AMCs), Orphan drug legislation (e.g. the US Orphan Drug Act of 1983) and Priority Review Vouchers issued under the Food and Drug Administration Amendments Act of 2007 (FDAAA).

These mechanisms have in general been conceptualized and implemented by the developed world and either international or philanthropic organizations. They do not take full advantage of the brainpower and infrastructure existing in middle-income developing countries or in some innovative developing countries (IDCs) [7] such as Brazil, where considerable progress has recently been made in defining and implementing a national policy for science, technology and innovation in health [8],[9],[10].

Research and development on neglected diseases is one of the key strategic areas of Brazil's priority agenda for health research [8],[11]. In 2005 the Ministry of Health together with the Ministry of Science and Technology, through their funding agencies DECIT (Department of Science and Technology, http://dtr2001.saude.gov.br/sctie/decit/index.htm) and CNPq (National Council for Scientific and Technological Development, http://www.cnpq.br/english/cnpq/index.htm), launched a joint Program to support research, technological development and innovation on six diseases that disproportionately hit poor and marginalized populations in Brazil: dengue, Chagas disease, leishmaniases, leprosy, malaria and tuberculosis. In 2008 schistosomiasis was added to the list and a 2nd call for applications instituted (http://www.cnpq.br/editais/ct/2008/034.htm). For additional detais on this DECIT/CNPq Program see Serruya et al [11],[12].

As equity and capacity building were considered critical components of the Program, it was decided to invest at least 30% of the financial resources in the three Brazilian geographic Regions where these diseases are still prevalent (North, Northeast and Center-West). Since the scientific productivity related to neglected diseases is less than in other areas of health sciences and several institutions located in these Regions are still maturing, traditional indicators such as number of scientific articles and impact factor of the journals where they were published would be of only limited value. We therefore decided to develop new approaches and criteria based on social network analysis [13],[14],[15],[16], to allow for a fair and efficacious allocation of resources without losing sight of scientific standards.

## Methods

### Data mining

Publications by Brazilian authors on the seven diseases were retrieved as raw data files from the ‘Web of Knowledge’ database of the Institute for Scientific Information (ISI), a database that lists the full addresses of all authors of every paper. Queries were made in ‘advanced search’ mode directed simultaneously at the country name *and* at words in the titles of the papers, e.g. [CU = Brazil AND TI = (Chagas OR cruzi)] to retrieve papers with at least one researcher from Brazil among the authors and having “Chagas disease” or “*Trypanosoma cruzi*” in the title.

### Standardization of names and addresses of authors and institutions

The ISI raw data files were imported into the text-mining software VantagePoint (http://www.thevantagepoint.com) with the appropriate ISI filters. A process of standardization was carried out to bring together the various different names of a particular author or institution [17] and VantagePoint thesauri for names and addresses were created in order to process additional name and address lists.

### Network assembly, visualization and analysis

Co-occurrence matrices of authorship data were built into VantagePoint and exported to UCINET software for social network analysis [18]. A co-occurrence matrix shows the number of records in the dataset containing two given list items. Symmetrical, co-occurence matrices (also called ‘adjacency matrices’) were created using the same set of authorship data in rows and columns in order to map co-authorships between authors (authors×authors matrices) or institutions (institutions×institutions matrices). For additional details on the use of matrices in social network analysis see for instance Chapter 3 of Scott [19], “Handling Relational Data”. Networks were assembled, visualized and analyzed for several parameters such as network components and cut-points with the softwares NetDraw or Pajek [20] which are embedded in the UCINET package.

## Results

### Publications on seven neglected diseases by Brazilian authors in peer-reviewed international journals

**Figure 1:**
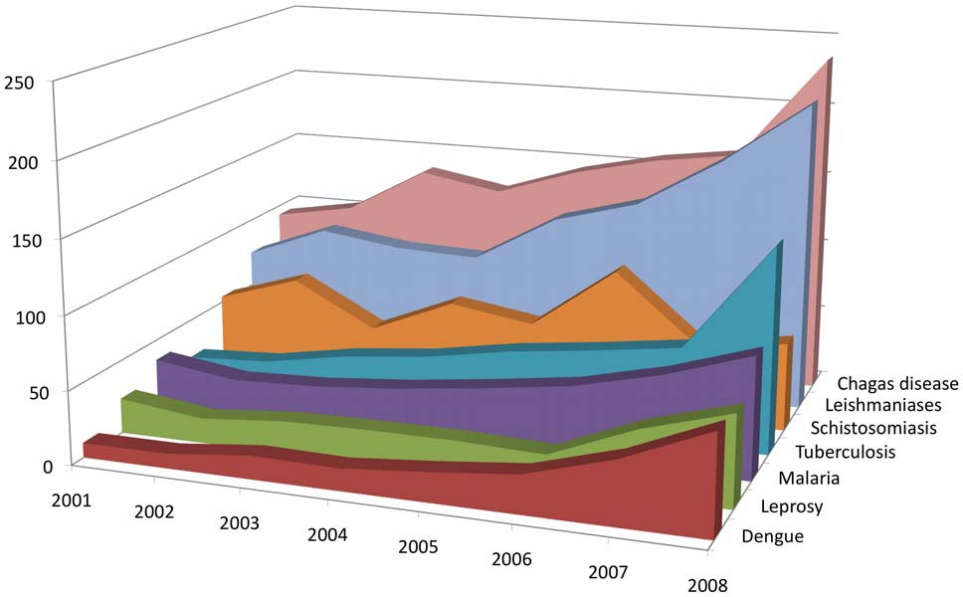
Evolution of publications by Brazilian authors on the seven neglected diseases covered by the DECIT/CNPq Program. Publications were retrieved from Thomson Reuters' ISI Web of Knowledge using the queries described in Methods. The recent increase in the scientific productivity occurs in all diseases with the exception of schistosomiasis, which was not included in phase I of the Program.

The scientific environment where the Program is based and operates can be assessed analyzing the scientific productivity of Brazilian authors and institutions in peer-reviewed international journals. Table 1 and Figure 1 display that it varies widely among the diseases covered by the Program, being for instance 5-fold greater for Chagas disease and leishmaniases as compared with dengue and leprosy.

**Table 1:** Publications by disease and year by Brazilian authors for the 2001–2008 period.

Year	Dengue	Leprosy	Malaria	TB	Schisto	Leish	Chagas	Total
2001	10	24	36	23	54	75	93	315
2002	10	17	28	26	72	97	103	353
2003	16	22	30	36	42	89	135	370
2004	15	23	35	42	66	87	125	393
2005	20	23	42	52	57	120	144	458
2006	26	21	50	59	100	136	157	549
2007	43	45	64	67	52	171	165	607
2008	68	62	83	141	61	214	236	865
Totals	208	237	368	446	504	989	1158	3910

The following words or combination of words were used to retrieve publications from Thomson Reuters' ISI Web of Knowledge database by querying the titles of the articles: dengue; leprosy OR leprae; malaria* OR vivax OR falciparum; tuberculosis; schistosom* OR mansoni; leishm* OR antileishm*; Chagas OR cruzi.

### Visualization and analysis of co-authorship networks

Co-authorship network analyses were carried out at several stages of the two phases of the Programme: Phase I included six diseases, funding projects during the biennium 2007–2008 and the ongoing Phase II addresses seven diseases during the 2009–2010 biennium. We decided to focus our attention on network components and network cut-points, basic elements of social network analysis [19],[21] that generate visual information readily useful for Program managers and decision-makers. In this way we emphasized the generation of graphical displays over a purely quantitative, numerical analysis.

### Components

**Figure 2:**
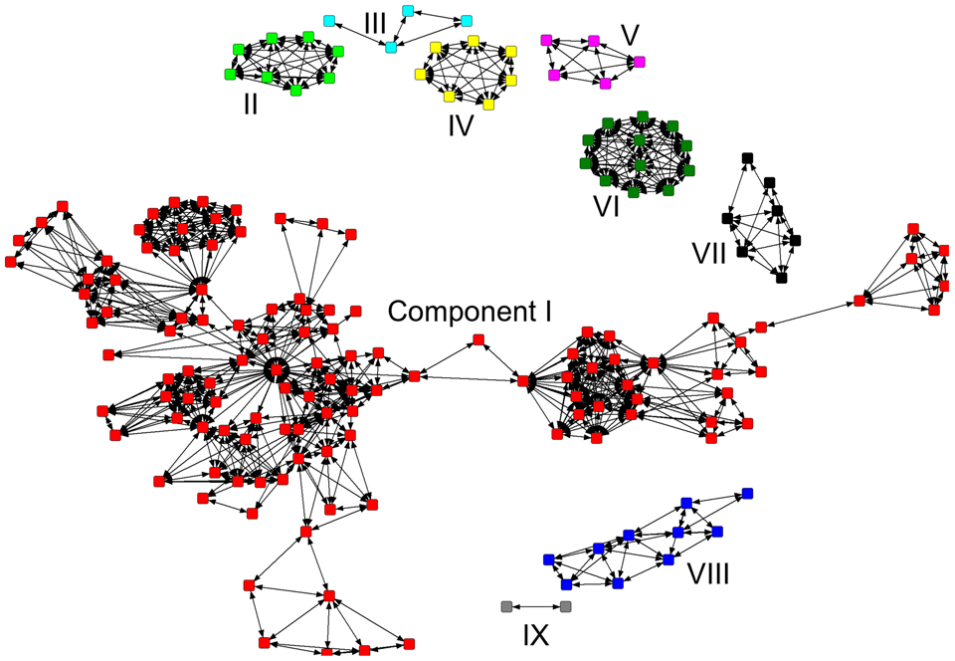
Components, dengue co-authorship researchers network, 2001–2008. Each square represents one author and each line connecting two authors indicates the presence of at least one publication they have co-authored. Components are defined by the observed co-authorships for the period under analysis; authors in one component have no publication in collaboration with authors of other components. This dengue network includes 174 authors with 2 or more papers published during the 2001–08 time span and contains nine components (labeled I–IX). The areas of work of the minor components can be inferred from the analysis of the most frequent keywords listed in their publications: Component II: blood donors; Brasil; dengue virus nucleic acid test; dengue virus RNA; detection; development; high-throughput blood screening; Honduras; prototype transcription-mediated amplification assay. Component III: social representations; control activities; dengue vectors; plant vases; relationships; residents; Sao Paulo State. Component IV: classical dengue fever; dengue shock syndrome; liver transplant recipient; liver transplantation. Component V: antiviral activity; algal-derived DL-galactan hybrid; carrageenans; chemical structure; *Meristiella gelidium*; sulfated polysaccharides; virus serotype; vitro dengue virus infection. Component VI: high dosages; dengue hemorrhagic fever; gamma globulin; immunoglobulin; serious thrombocytopenia; treatment. Component VII: apoptosis; dengue virus fusion peptide; dengue virus infection; energy charge; HepG2 cell; lipid membrane; membrane fusion; metabolism; mitochondrial dysfunction; oligomerization; partition. Component VIII: *Aedes aegypti*; dengue Control Program; dengue transmission; environmental variables; health agents' work; population adherence; spatial analysis; spatial correlation.

A component of a network is a portion of the network in which all actors are connected, directly or indirectly, by at least one tie (one co-authorship in the present work) [21]. Fig. 2 shows the component analysis of the 2001–2008 dengue co-authorship network, where 172 authors are distributed among 9 components, each one addressing in isolation its own set of specific, complementary or overlapping research topics and subjects.

### Cut-points

**Figure 3:**
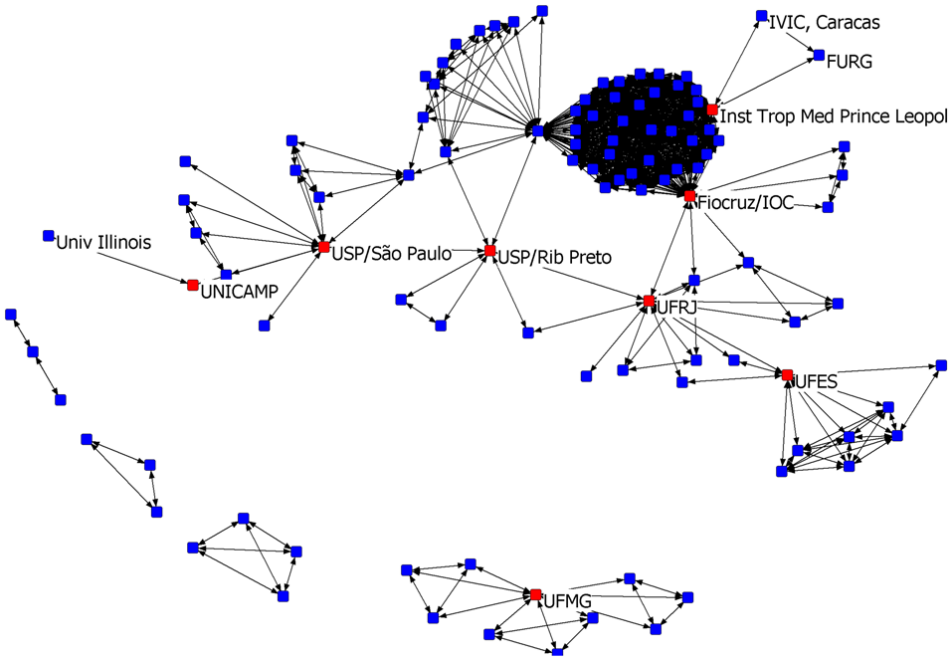
Cut-points, tuberculosis co-authorship institutions network, 2006–2007. Each node represents an institution that published at least one article in the biennium 2006–07. Nodes in red are institutions that function as cut-points in this network: the Oswaldo Cruz Institute at the Oswaldo Cruz Foundation (Fiocruz/IOC) in Rio de Janeiro; the Prince Leopold Institute of Tropical Medicine or Institute of Tropical Medicine (ITM) in Antwerp; the Federal University of Espírito Santo (UFES); the Federal University of Minas Gerais (UFMG); the Federal University of Rio de Janeiro (UFRJ); the University of Campinas (UNICAMP); the University of São Paulo in Ribeirão Preto (USP/Ribeirão Preto); and the University of São Paulo in São Paulo (USP/São Paulo). The role of ITM as a cut-point derives from the article by da Silva et al, co-authored by seven authors from ITM, the Federal University of Rio Grande, RS, Brazil (FURG) and the Instituto Venezolando de Investigaciones Cientificas, Caracas, Venezuela (IVIC) [36]; in a similar way the paper by Pasqualoto et al links the University of Illinois to the TB network through the cut-point UNICAMP [37]. The large agglomerate of nodes is due to the article by Brudey et al [38] which has 66 authors from 42 institutions, two of them acting as cut-points (Fiocruz/IOC and ITM).

A cut-point of a network is an actor (author or institution in our case) whose removal would increase the number of components by dividing the sub-graph into two or more separate subsets between which there are no connections. Cut-points are therefore pivotal points of articulation between the elements that make up a component [19]. The role of cut-points is exemplified in Fig. 3, which shows the 2006–2007 tuberculosis institutional co-authorship network with the cut-point nodes labeled and identified as red squares. In this network, for instance, the removal of the cut-point “Inst. Trop. Med. Prince Leopold” would disconnect FURG and IVIC from the network and the removal of the cut-point “UNICAMP” would do the same for the University of Illinois. The visualization of this network also demonstrates the power of graphic displays to rapidly detect and emphasize unique features of a given network. In this figure, the large agglomerate of nodes at the upper left immediately stands out, drawing one's attention to the presence of a publication involving a large number of coauthors and their institutions, an indicator of projects involving global networks.

## Discussion

### Evaluation of scientific productivity on neglected diseases: the need for new strategies and approaches

Traditional scientific production indicators routinely adopted as criteria for evaluating scientific proposals and research funding programs, such as the number of publications in a given period of time and impact factor or H-index [22], have intrinsic shortcomings [23],[24] and are of limited value beyond ‘Mode 1’ of knowledge production (disciplinary, primarily cognitive, context) [25] or when the publication output of the work field or the scientific community under consideration is of small size. In fact a ‘Catch-22’ type challenge (a no-win situation or a double bind dilemma, see http://en.wikipedia.org/wiki/Catch-22) arises when considering these indicators to select candidates eligible for capacity building purposes, as the researchers and institutions most in need of support are exactly those who have a modest scientific curriculum or performance. Traditional evaluations therefore become a real barrier to career progress or towards institutional development.

The management of the DECIT/CNPq Program, having received the double mandate to adhere to high scientific standards *and* strengthen capacity in the less developed Regions of the country, as two pillars of the initiative, realized that new strategies and indicators would be needed. The 2001–2008 survey of publications shown in Tables 1 and 2 well illustrates some of the challenges the Program would face, for instance: (i) three out of the four most active research communities (Chagas disease, schistosomiasis and tuberculosis) are located in the developed South and Southeast of Brazil, far from the target Regions for capacity building and (ii) dengue, a disease that has caused serious problems for public health in recent years, has been addressed by one of the smallest scientific communities and needs ‘fast-track’ capacity building actions.

**Table 2:** Geographic distribution of key network institutions conducting R&D on the neglected diseases of the DECIT/CNPq Program, 2001–2007.

Disease	Top-10′ institutions by number of publications			Additional N/NE/CW institutions identified by their cut-point location in co-authorship networks
	N/NE/CW	S/SE	Foreign	
Chagas disease	0	10	0	Hospital Anis Rassi; UFPE
Dengue	4	6	0	CEPEM
Leishmaniases	2	8	0	UFGO; UFRN
Leprosy	1	7	2*	UFCE
Malaria	4	6	0	CEPEM; UFBA
Schistosomiasis	0	7	3**	CCBi; CPqAM; UFBA
Tuberculosis	0	10	0	None
Total	11	54	5	The above 9 additional institutions were identified by the cut-point criterion
	70			

For each disease we mapped the ten institutions ranking higher in total number of publications on neglected diseases in international peer-reviewed journals having at least one Brazilian author (the ‘top-10’ institutions in Chagas, the ‘top-10’ in dengue, etc.). The majority of the ‘top-10’ Brazilian institutions are located in the more developed regions of Brazil (South/Southeast, 54 institutions) and not where most of these diseases are endemic (North/Northeast/Center-West, 11 institutions). Co-authorship network analysis allowed the identification of 9 *additional* key institutions from these less developed regions based on another criterion: their critical role in contributing to network structure, function and sustainability due to their location at ‘cut-points’ of the networks. *Brazilian cut-point institutions at N/NE/CW*: Aggeu Magalhães Research Center (CPqAM), Oswaldo Cruz Foundation (Fiocruz), Recife, Pernambuco; Center for the Biological Sciences, Federal University of Alagoas, Maceió, Alagoas (CCBi); Federal University of Bahia (UFBA); Federal University of Ceará (UFCE); Federal University of Goiás (UFGO); Federal University of Pernambuco (UFPE); Federal University of Rio Grande do Norte (UFRN); Hospital Anis Rassi, Goiania, Goiás; Tropical Medicine Research Center, Porto Velho, Rondonia (CEPEM).

*Foreign institutions collaborating with Brazilian authors*: * London School of Hygiene and Tropical Medicine; University of Tubingen; ** University of Glasgow; Purdue University; George Washington University.

Two social network analysis tools proved to generate particularly valuable information for the strategic management of the Program, the identification of components and cut-points of the co-authorship networks:

### Identification and characterization of network components

Component analysis generates a picture of the overall network structure, revealing how fragmented it is and therefore providing valuable information on its status and opportunities for strategic management. As shown in Fig. 2 for the dengue researcher's network, the analysis of the work areas of the nine individual components, based on article keywords, suggested for instance, a collaboration between component III and VIII, as their researchers were all working on dengue vector control but did not engage in formal collaborations.

### Identification and characterization of network cut-points

The identification of network cut-points became a very important analytical tool for the management of the Program, particularly in relation to its capacity building/strengthening mandate. As the majority of institutions in the less developed Regions still need to mature, a selection based exclusively on scientific productivity would place them at a clear disadvantage in comparison with sister institutions from the developed Southeast and South. We realized that institutions acting as network cut-points were critical key players as they were responsible for keeping several institutions from these Regions in the loop and should therefore be considered as fundamental partners for training, capacity building and institutional strengthening. This reasoning is supported by work in other fields that made evident the importance of scientists who play roles as brokers for communications among others [14], the function of nodes critically involved in connecting or bridging modular subregions of a network [26] or the cruciality of ‘creative elements’ in cells, social networks and ecosystems [27].

Table 2 shows that by adopting this cut-point criterion to help the selection of institutions worth strengthening, nine ‘cut-point institutions’ could be added to the eleven ‘top-10 institutions’ identified by classical high-productivity criteria. The Program could therefore double the number of potential investment targets in the North, Northeast and Center-West Regions with objective science-based parameters: the traditional, productivity-based indicators together with the new ones derived from the network analysis proposed in this article.

### How were the new approach and indicators put into action?

The Program was shaped to operate in ‘Mode 2’ of knowledge production (broader, transdisciplinary social and economic contexts) [25] as its mission goes beyond academic goals to also address capacity building, institution strengthening, product development, disease control and public health. In Brazil's national health system (SUS - *Sistema Único de Saúde*) the participation of civil society and communities is assured at all levels of government - federal, state and municipal [28]. The process leading to the prioritization of R&D on neglected diseases, which made possible the launching of the DECIT/CNPq Program and set its main objectives and goals, involved strong participation of these key stakeholders e.g. at the National Health Council (http://conselho.saude.gov.br/apresentacao/index.htm) and at the 2nd National Conference on Science, Technology, and Innovation in Health held in 2004 which involved 15,000 participants [8].

Mobilizing the scientific community, disease control managers and policy/decison-makers to collaborate under the umbrella of this initiative required a sort of ‘cultural change’ from everyone involved. For this purpose the process adopted by the Program included: (i) Organizing priority setting workshops with equal representation by researchers, policy/decision-makers and managers interested in the seven diseases of the Program; (ii) Adopting guiding principles such as burden of disease and classical/network-based science indicators as the basis for workshop agendas and discussions; (iii) Structuring these workshops on disease-specific working groups with equal representation of policy/decision-makers, managers and scientists of high productivity and/or affiliated to network cut-point institutions; (iv) Mobilizing the participation of scientific communities through ‘Call for Applications’ based on the recommendations of the working groups and published in the websites of the funding agencies; (v) Peer reviewing of the proposals taking into account the need to allocate a minimum of 30% of the funds to projects submitted by principal investigators affiliated to institutions in the North, Northeast and Center-West Regions.

Fig. 1 suggests that the DECIT/CNPq Program has been successful in stimulating scientific productivity on the six diseases in its first phase which did not include schistosomiasis as one of the targets. The future assessment of the full impact of the two phases, however, will need a thorough in-depth evaluation exercise based on input, output, outcome and impact indicators addressing scientific, technological and public health goals. Co-authorship network analysis has been employed to evaluate scientific journals [29],[30], institutions [31] and collaboration patterns in specific scientific fields [17]. The innovative contribution brought by this analytical approach during the shaping and implementation of the Program will be expanded and become critical when assessing the evolution, performance and robustness of the networks involved.

Our results also suggest that co-authorship network analysis could become an important tool for international organizations or partnerships targeting the elimination or eradication of diseases, providing science-based information relevant to strategic analysis and planning. Lessons from past eradication campaigns demonstrated the importance of maximizing the utilization of scarce human and financial resources, functioning within existing health service structures and encouraging research at all levels [32]. Applied to today's planned efforts towards the elimination/eradication of malaria [33],[34] or neglected tropical diseases [35], these lessons would mean identifying and engaging health services, researchers and institutions from developed and endemic countries, an immense challenge that co-authorship network analysis could help address, providing a substantial contribution to global health.

## Supporting Information

Alternative Language Abstract S1 Spanish translation of the abstract by Walter Alejandro I. Casas.
(0.03 MB PDF)
