# Supplementary material for: Co-authorship Network Analysis: A Powerful Tool for Strategic Planning of Research, Development and Capacity Building Programs on Neglected Diseases
Source: PLoS Negl Trop Dis. 2009 Aug 18;3(8):e501. doi: 10.1371/journal.pntd.0000501 (PMC2721762; doi:10.1371/journal.pntd.0000501)
Supplement: Alternative Language Abstract S1 — Spanish translation of the abstract by Walter Alejandro I. Casas. (0.03 MB PDF) [file pntd.0000501.s001.pdf]

Resumen

Antecedentes. Fueron necesarios nuevos abordajes e instrumentos para dar apoyo a la planificación estratégica, implementación y administración de un Programa puesto en marcha por el Gobierno Brasileño para financiar la investigación, el desarrollo y la construcción de capacidades en enfermedades tropicales desatendidas, focalizado fuertemente en las regiones Norte, Noreste y Centro-Oeste del país donde dichas enfermedades son prevalentes.

Metodología/Principales Resultados. Con base en datos demográficos, epidemiológicos y de carga de morbilidad fueron seleccionadas siete enfermedades por el Ministerio de Salud como objetivos de la iniciativa. Publicaciones de investigadores brasileños sobre estas enfermedades fueron extraídas de bases de datos internacionales; analizadas y procesadas con instrumentos de mineración de texto con el propósito de estandarizar los autores e instituciones por nombres y direcciones. Redes de coautoría basadas en estas publicaciones fueron montadas, visualizadas y analizadas con los paquetes de Programas informáticos sobre análisis de redes sociales. La visualización y análisis de las redes generó una nueva información posibilitando un mejor diseño y planificación estratégica del Programa, permitiendo a los tomadores de decisión caracterizar los componentes de la red por área de trabajo, identificar instituciones así como los autores que desempeñan papeles principales como ejes centrales o localizados en puntos de corte críticos de la red, y detectar fácilmente a los autores e instituciones que participan en grandes redes de colaboración científica internacionales.

Conclusiones/Importancia. Los criterios tradicionales, usados para supervisar y evaluar los proyectos de investigación o los Programas de I+D tales como la productividad de los investigadores y el factor de impacto de publicaciones científicas, tienen valor restringido cuando están dirigidos a áreas de baja productividad o que envuelven regiones endémicas donde los recursos humanos son limitados. El análisis de redes sirvió para generar nueva y valiosa información relevante para la planificación estratégica, implementación y supervisión del Programa. Esto posibilitó un papel más proactivo de las agencias de financiación, en lo referente a metas de salud pública y equidad, objetivos de fortalecimiento de capacidad científica y un compromiso más consistente de autores e instituciones de regiones endémicas, basado en criterios innovadores y en parámetros fundamentados en datos científicos objetivos.

Resumen del autor

La selección y priorización de los proyectos de investigación es siempre un gran desafío, particularmente cuando tratan sobre enfermedades tropicales desatendidas, donde las comunidades científicas son relativamente pequeñas, el financiamiento es generalmente limitado, y la disparidad de la ciencia y capacidad tecnológica entre los diversos países y regiones es enorme. Cuando el Ministerio de Salud conjuntamente al Ministerio de Ciencia y Tecnología de Brasil decidieron lanzar un Programa de I+D para enfermedades desatendidas, sobre el cual hubo una expectativa de inversión de por lo menos 30% de los recursos del programa en instituciones y autores de las regiones más pobres del Brasil, se hizo manifiesto para nosotros que serían necesarios nuevas estrategias, abordajes e instrumentos. El análisis de redes sociales de las redes de coautoría [1,2,3,4] es uno de los nuevos abordajes que estamos explorando para desarrollar nuevos instrumentos que ayuden conjuntamente a los formuladores de política/tomadores de decisión y a la academia para planificar, ejecutar, supervisar y evaluar las inversiones en esta área. Este artículo representa nuestra primera incursión en este emocionante campo.
